# Isolation and characterization of a *Vibrio owensii* phage phi50-12

**DOI:** 10.1038/s41598-022-20831-2

**Published:** 2022-09-30

**Authors:** Ling-Chun Lin, Yu-Chuan Tsai

**Affiliations:** Masters Program in Biomedical Sciences, School of Medicine, Tzu Chi University, No. 701, Sec. 3, Zhongyang Rd., Hualien, 97004 Taiwan

**Keywords:** Microbiology, Diseases

## Abstract

*Vibrio owensii* is a widely distributed marine vibrio species that causes acute hepatopancreatic necrosis in the larvae of *Panulirus ornatus* and *Penaeus vannamei,* and is also associated with Montipora white syndrome in corals. We characterized *V. owensii* GRA50-12 as a potent pathogen using phenotypic, biochemical, and zebrafish models. A virulent phage, vB_VowP_phi50-12 (phi50-12), belonging to the N4-like *Podoviridae,* was isolated from the same habitat as that of *V. owensii* GRA50-12 and characterized. This phage possesses a unique sequence with no similar hits in the public databases and has a short latent time (30 min), a large burst size (106 PFU/infected cell), and a wide range of pH and temperature stabilities. Moreover, phi50-12 also demonstrated a strong lysis ability against *V. owensii* GRA50-12. SDS-PAGE revealed at least nine structural proteins, four of which were confirmed using LC–MS/MS analysis. The size of the phi50-12 genome was 68,059 bp, with 38.5% G + C content. A total of 101 ORFs were annotated, with 17 ORFs having closely related counterparts in the N4-like vibrio phage. Genomic sequencing confirmed the absence of antibiotic resistance genes or virulence factors. Comparative studies have shown that phi50-12 has a unique genomic arrangement, except for the well-conserved core regions of the N4-like phages. Phylogenetic analysis demonstrated that it belonged to a group of smaller genomes of N4-like vibrio phages. The therapeutic effect in the zebrafish model suggests that phi50-12 could be a potential candidate for application in the treatment of *V. owensii* infection or as a biocontrol agent. However, further research must be carried out to confirm the efficacy of phage50-12.

## Introduction

The genus *Vibrio* comprises a large number of bacterial species that are widespread in coastal oceans^1^. In recent years, many new marine *Vibrio* species have been isolated, identified, and analyzed because of their unique physiological and ecological characteristics^2,3^. Among them, the Harveyi clade, which contains many species related to fishery or coral pathogenicity^4–8^, has attracted much attention. For example, *Vibrio harveyi* is not only an important pathogenic bacterium that causes fish and crustacean infections, but also is an important research model for studying quorum sensing mechanisms in the cooperative behavior of bacteria^9^. The Harveyi clade in the *Vibrio* group contains five major marine pathogenic bacteria^10^, *V. harveyi*, *V. campbellii*, *V. owensii*, *V. jasicida*, and *V. rotiferianus*. They are well-recognized aquatic animal pathogens, but misclassification is common due to similarities in their rRNA sequences and phenotypes^11,12^.

This study majorly focuses on *V. owensii* which was officially identified and published by Australian microbiologists Leigh Owens in 2010, and the published strain was isolated from diseased shrimp in lobster farms^13^. *V. owensii* is a gram-negative, facultative anaerobic bacterium with a terminal flagellum. It has been recognized as a marine animal pathogen. A previous study reported that *V. owensii* can cause infection of larval cultures in the breeding stock of *Panulirus ornatus*, resulting in serious economic losses^14^. A study of Artemia (brine shrimp) mixed with *V. owensii* DY05, used as food for lobster larvae found that the cumulative lethality rate reached 84–89% after 72 h^14^. Anatomical analysis of the tissue showed that *V. owensii* was attached to the hepatopancreas and caused necrosis of epithelial cells (Acute hepatopancreatic necrosis disease, AHPND). This showed that *V. owensii* DY05 is a virulent enteric bacterium of *Panulirus ornatus*. However, necrotic infection of the hepatopancreas in lobster seedlings caused by *V. owensii* is not a special case of DY05, and other researchers have isolated a virulent *Vibrio owensii* strain SH-14 as the causative agent of AHPND in cultured shrimp^15^. Genome analysis revealed that this strain contained a plasmid. This plasmid was sequenced, analyzed, and found to be similar to the plasmid contained within strain *V. parahaemolyticus*, which has been reported to cause acute hepatopancreatic necrosis disease in the past^16^. These plasmids are 99.1% similar and are therefore considered to be associated with acute hepatopancreatic necrosis^17^. Studies have also shown that *V. owensii* is associated with Montipora white syndrome in the Hawaiian Islands^8^. Coral bleaching has gained considerable attention in Taiwan recently^18^. Previous studies suggest that *V. owensii* can infect aquatic animals, but its toxicity mechanism remains unclear.

Overuse of antibiotics in aquatic farms increases antibiotic resistance^19^. Phages have gained attention as biocontrol or therapeutic agents^20^. To date, only a few phages have been reported against *V. owensii*^21^. In this study, we used *V. owensii* GRA50-12 as the host to isolate and identify phages that can be used to treat *V. owensii* infection.

## Results and discussion

### Biological characteristics of *V. owensii* GRA50-12

*V. owensii* GRA50-12 was isolated from a coral-associated macroalgae symbiome in the intertidal zone of eastern Taiwan. The whole genome sequence obtained using shotgun sequencing has been published and deposited at DDBJ/EMBL/GenBank under accession numbers BBPJ01000001 to BBPJ01000051. Using Illumina Solexa as the sequencing platform, with 652× genome coverage, the current status of this draft genome was noted as “high quality draft”^22^. According to the Genome Neighbors report provided by NCBI (Supplementary Table S1), the most closely related species are OCN002^23^ with 90% sequence identity, strain bablab_jr007^24^ with 89.8% identical sequence, and one completely sequenced genome XSBZ03^25^ with 89.5% sequence identity. All of these have been isolated from diseased corals. This suggests that some *V. owensii* shares similar genetic composition in certain ecological niches. When grown on TSB3S routine culture plates, GRA50-12 showed smooth and opaque colonies (Supplementary Fig. S1). Pathogenicity is often attributed to many secretory substances, such as proteases, hemolysins, lipases, or bacteriocin-like substances. Clinical isolates of *V. parahaemolyticus* often produce hemolysins on Wagatsuma agar plates^26^. Hemolytic activity tests were performed on Wagatsuma agar plates. Our results demonstrated a clear zone surrounding the bacteria (Supplementary Fig. S1), indicating the hemolytic ability of *V. owensii* GRA50-12. This result is similar to that of clinical isolates of *V. parahaemolyticus* which produce heat-stable hemolysin^26^. The API20E test revealed that GRA50-12 has biochemical characteristics identical to *V. owensii* pathogenic strains DY05 and 47666-1 with the result A−/L+/O+ (Supplementary Table S2)^14^. *V. owensii* strain DY05 has been demonstrated to be the etiological agent in larviculture of the ornate spiny lobster (*Panulirus ornatus*), which causes rapid and reproducible larval mortality with pathologies similar to those seen during epizootics.

**Figure 1:**
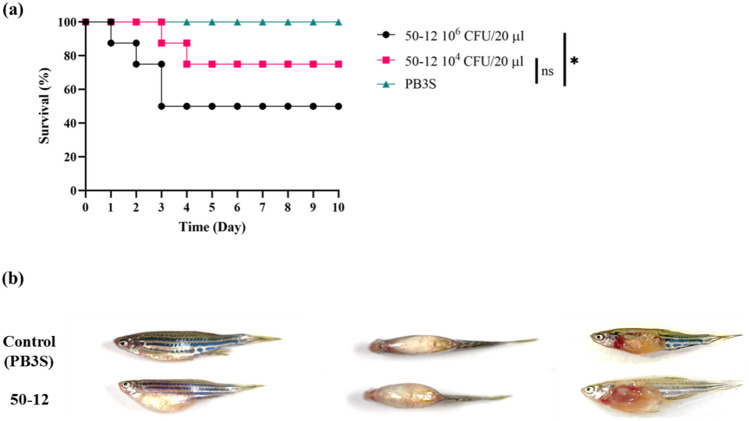
Pathogenicity of *V. owensii* GRA50-12 was confirmed by using zebrafish as a model. (**a**) Three groups (8 fishes/group) were considered, and their survival rates were measured after intraperitoneal injection of *V. owensii* GRA50-12 cells (2.2 × 10^6^ CFU/20 µl, black line; 2.2 × 10^4^ CFU/20 µl, red line) or PB3S (PBS with 3% NaCl) as control (green line). The X-axis represents the time post-infection and the Y-axis represents the survival percentage of the zebrafish. (**b**) The disease symptoms in *V. owensii* GRA50-12 infected zebrafish. Control represents PB3S buffer injected fish; thus, no symptoms were observed. 50-12 represents zebrafish injected with GRA50-12 and these showed swollen abdomen and bleeding at body surface (left: side view; middle: top view; right: abdomen anatomy). The anatomical analysis showed swollen and bleeding intestines. The significance of the differences between groups was performed by Log-rank and Gehan–Breslow–Wilcoxon test in GraphPad Prism 9. “*” indicates p < 0.05.

Zebrafish were used as a model to confirm the pathogenicity of GRA50-12. Several pathogenic *Vibrio* species used in zebrafish models have been previously described, and are highly relevant to real fish models^27^. When 2 × 10^6^ CFU/20 µL of GRA50-12 was injected into zebrafish through the cloaca, fish death reached LD_50_ after 3 days (Fig. 1a). The diseased fish showed distinguished symptoms with a swollen abdomen, and anatomical analysis showed swollen and bleeding intestines (Fig. 1b). These results suggest our zebrafish model showed that GRA50-12 causes fish diseases and the model is useful for demonstrating the effectiveness of phages in controlling the disease.

### Phage morphology and biological properties

**Figure 2:**
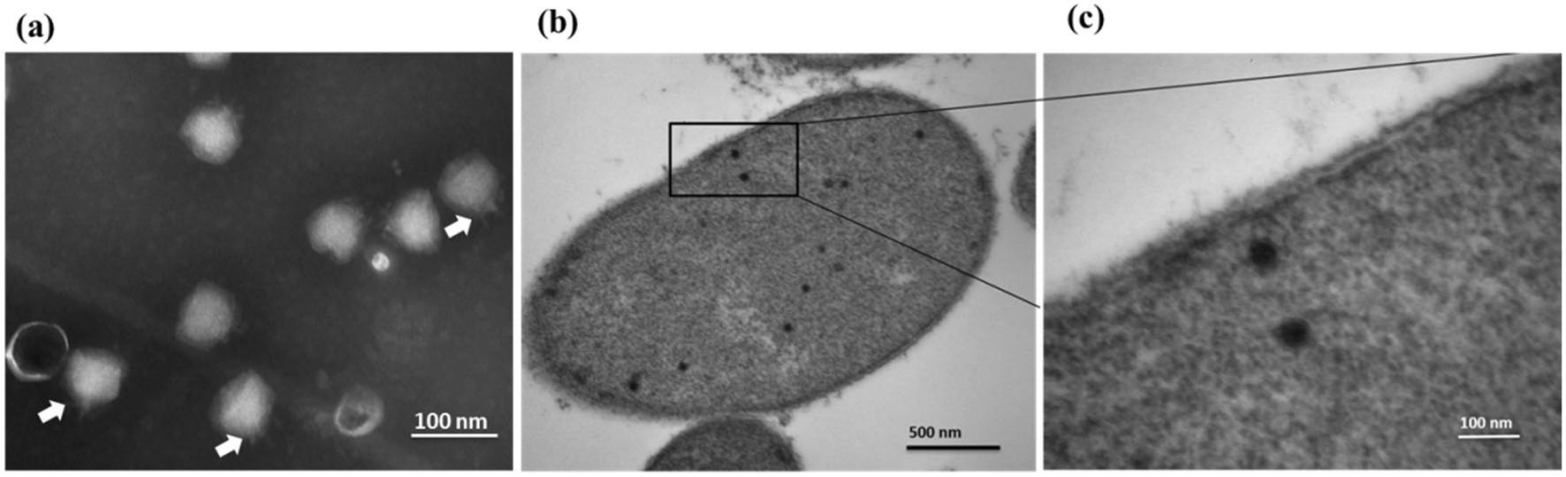
Morphology of phi50-12. (**a**) Electron micrograph of phi50-12 demonstrates that it resembles podoviruses with short tails; arrow heads indicate the tails of phi50-12. (**b**) Electron micrograph of thin section reveals phi50-12 virions inside the cell and (**c**) the loss of cell surface integrity during the release of virions.

Vibrio phage phi50-12 was isolated from a water sample from the intertidal zone around the location where the host *V. owensii* GRA50-12 was isolated. The plaques of phi50-12 formed on the lawns of GRA50-12 displayed a halo surrounded by a clear zone which increased in size as time progressed from day 1 to day 3 (Supplementary Fig. S2). This might indicate depolymerase activity, as previously reported^28^. The isolated phage was designated as phi50-12 based on its host origin. Cesium chloride-purified phage particles were concentrated, and their morphology was examined by TEM (Fig. 2a). Morphologically, phi50-12 showed visibly short tails with icosahedral capsid heads; it resembled podoviruses. The size of the capsid is approximately 70 nm. Electron micrographs of ultrathin sections demonstrated mature phi50-12 virions that were released along the surface of *V. owensii* GRA50-12; at sites where virus particles were released, membrane integrity was lost eventually resulting in cell lysis (Fig. 2b,c, and Supplementary Fig. S3).

The adsorption of phi50-12 reached 60% after 3 min, and no free unadsorbed phages were detected until 21 min post-infection (Supplementary Fig. S4). The latent interval between adsorption and the beginning of the burst was 30 min (Supplementary Fig. S4), and the average burst size was 106 PFU/infected cell. To evaluate the lytic activity of phi50-12, in vitro lysis was performed using different multiplicity of infection (MOI). The results indicated that phi50-12 could reduce or inhibit bacterial growth rapidly in the early growth stage, and that this was maintained for at least 10 h at different MOIs applied (Supplementary Fig. S5). Interestingly, with higher MOI, a low frequency of phage-resistant bacteria was generated, which switched their colony morphotype from opaque to translucent (Supplementary Fig. S6). Naturally, many vibrio species display opaque and translucent colony types, such as *V. parahaemolyticus* and *V. alginolyticus*^29,30^. Colony morphological changes involving physiological, biological, and virulence activity changes have been described^31,32^. In our study, translucent colony variants showed reduced sensitivity to phi50-12 (data not shown). The opaque and translucent phenotypes of *V. owensii* are plausibly caused by phase variation. A similar observation has been observed with *Bacteroides intestinalis* in a study examining infecting crAss-like phage crAss001^33^. However, further investigation is required to verify this hypothesis.

We determined the host ranges by spot tests and confirmed the results using plaque assays with a range of Harveyi-clade *Vibrio* species, including several strains of *V. parahaemolyticus*, *V. alginolyticus*, and *V. harveyi* (Table 1). The results showed that phi50-12 is a host stringent phage, and only *V. owensii* GRA50-12 and *Vibrio *sp. I55-5 (isolated from different types of algae) were infected by phi50-12. I55-5 was sequenced using 16S rRNA sequencing, and was identified as *V. harveyi*. The restricted host range was also observed in phages JA1 and VCO139 infecting *V. cholera*^34^.

**Table 1:** Host range of phi50-12 against *Vibrio* spp. in this study.

Species	Strains	Infectivity	Sources
*V. owensii*	GRA50-12	+	^22^
	051011B	−	Urbanczyk H^a^
*V. campbelli*	151112c	−	Urbanczyk H
*V. hyugaensis*	090810a	−	^60^
*V. jasicide*	090810c	−	^ 61 ^
*V.* spp*.*^#^			
	2-13	−	This study
	8-11	−	This study
	16-10	−	This study
	18-4	−	This study
	55-5	+	This study
	55-13	−	This study
*V. harveyi*			
	BCRC13812 (ATCC25919)	−	BCRC^b^
	0007	−	Chen CY^c^
	0200	−	Chen CY
	2233	−	Chen CY
*V. alginolyticus*^*#*^	15-1	−	This study
*V. parahaemolyticus*	93	−	Yu MS^c^
	10806	−	Yu MS

^a^University of Miyazaki, Miyazaki, Japan;

^b^Bioresource Collection and Research Center, Taiwan;

^c^Tzu Chi University, Hualien, Taiwan,

^#^Identification by selective medium TCBS and 16S rRNA sequencing.

**Figure 3:**
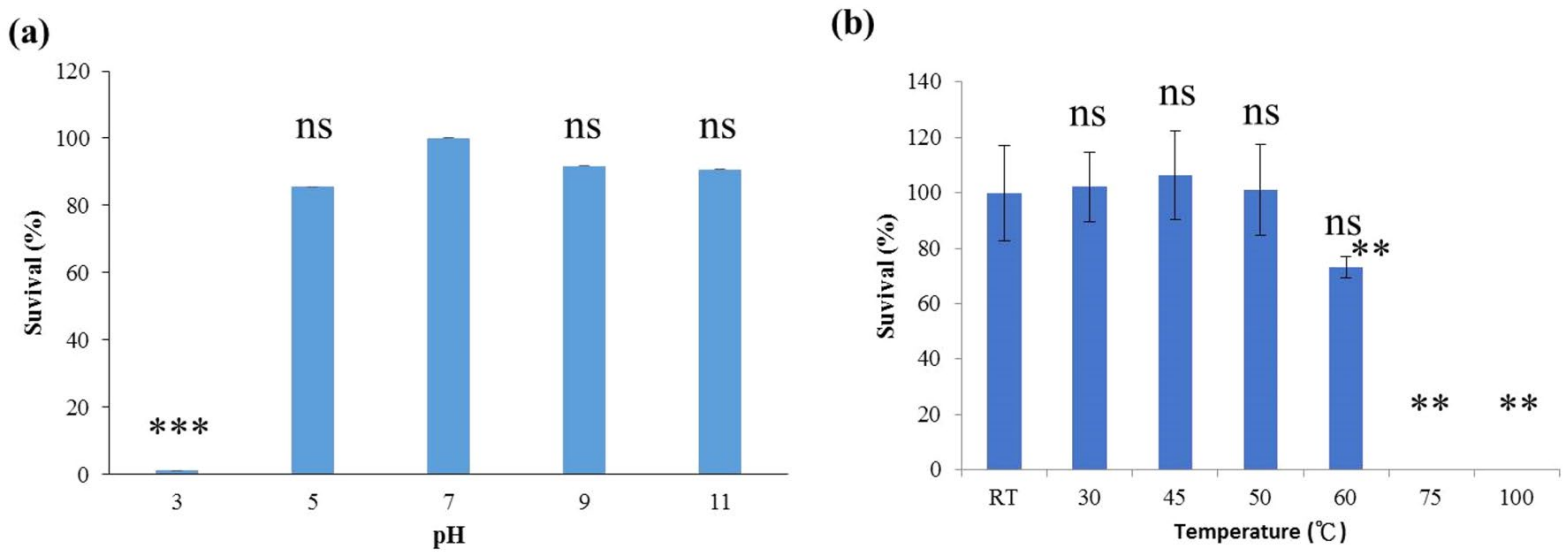
pH and thermal stability of phi50-12. (**a**) 10^6^ PFU of phi50-12 were subjected to various pH at 30 °C for one hour and titrated by plaque assay using a double-layer method; (**b**) phi50-12 stability (10^6^ PFU) under various temperatures for 1 h was measured by plaque assay. The survival rate was measured by comparison with initial phage number. The experiments were performed in triplicates and the data are shown as the mean ± SEM. Student’s *t* test was performed for significance. *Ns* no significance; **p < 0.01; ***p < 0.001.

Phi50-12 was subjected to several stresses to simulate environmental conditions. To evaluate the stability of viral particles, we assessed their infectivity at 15 min and 1 h of UV exposure, and at different salt concentrations, temperatures, and pH values for 24 h. The results showed that the particles were very sensitive to UV radiation, but had a high tolerance to salt fluctuation. In addition, phi50-12 showed a wider range of pH stability and lost activity at pH 3 (Fig. 3a). When exposed to various temperatures, the results showed phi50-12 to be stable at 4, 25, 37 °C but lost 18% activity at 60 °C and 100% at 75 °C (Fig. 3b).

### Genomic sequence and analysis

**Figure 4:**
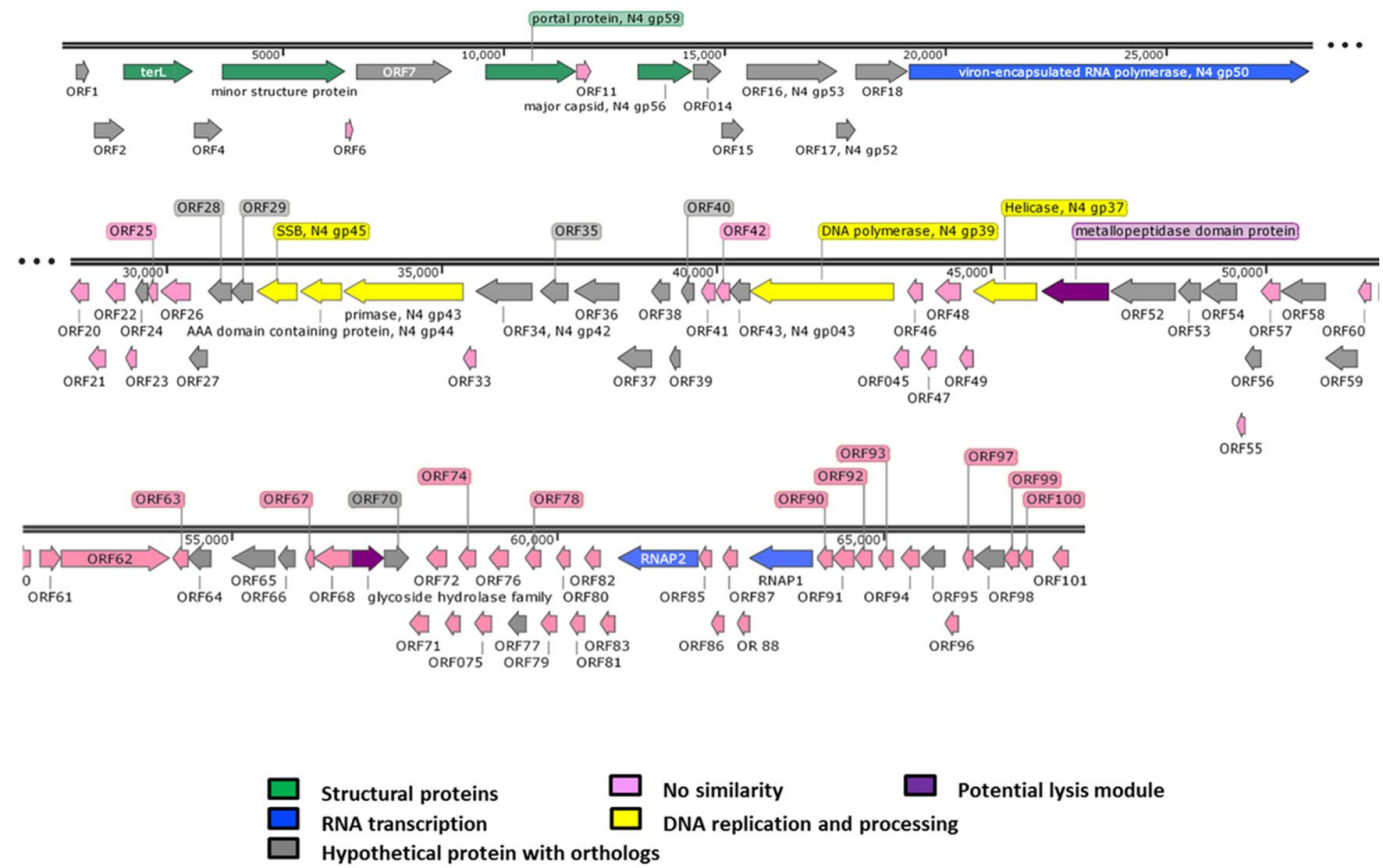
Genomic organization of phi50-12. Direction of the arrow represents transcription orientation. Colored box represents their functionality or uniqueness.

The genome size of phi50-12 was estimated to be 65–70 kb according to the results of pulse field gel electrophoresis (PFGE) (Supplementary Fig. S7). The purified phi50-12 DNA showed resistance to frequently used restriction enzymes, including BamHI, KpnI, PstI, SacI, SphI, and XbaI, indicating the existence of potential restriction modification systems. The complete genome sequence of phi50-12 is submitted to GenBank (Accession number MN584918.1), and the accurate genome size is 68,059 bp. The GC content of phi50-12 is 38.5%, which is lower than that of GRA50-12 (45.5%). Nucleotide sequence analysis using BlastN against the nt database revealed no related sequences in the public database. Using BlastP, 101 ORFs were annotated and grouped by functional modules (Fig. 4, Supplementary Table S3). Forty-nine ORFs matched homologs in the available databases, where seventeen of them have closely related counterparts in the N4-like vibrio phage (Supplementary Table S3). Similar to other N4-like phages, the majority of conserved homologs preserve the unique features, such as complete sets of RNA polymerases for orderly transcriptional expression, DNA replication, and capsid formation. No tRNA or tmRNAs were identified in phi50-12 using Aragorn^35^ and tRNAScanSE^36^, and no antibiotic resistance genes or virulence factors were found by ResFinder and VirulenceFinder^37^.

One of the unique features of N4 particle is the virion-encapsidated RNA polymerase (vRNAP). Genomic analysis of phi50-12 also revealed a large ORF (ORF19) with 3024 amino acids that matched N4 gp50 (vRNAP), which is responsible for early transcription^38^ and the facilitation of the injection of viral DNA into the host. Although smaller than other vRNAP in N4 and N4-like phages, the result of BlastP showed that phi50-12 vRNAP possesses a conserved domain, and was closely related to the vRNAP gene of Vibrio phage VCO139^34^. Phi50-12 encodes two RNA polymerases (RNAP1 and RNAP2); both of these are subunits of N4 RNA polymerase II; together with gp2 (another subunit), they transcribe the middle genes during the phage life cycle^39^. However, gp2 homologs were not detected in phi50-12. Many ORFs in phi50-12 have no known functions, and gp2 is likely to be functionally replaced by other ORFs. Similar to the arrangement of *Vibrio* N4-like phages, small insertions (~ 1.7 kb) between RNAP1 (ORF89) and RNAP2 (ORF84), have also been found in phi50-12^34^. ORFs between the two RNA polymerases were predicted to be hypothetical proteins with no similarity hits in the public database. Genes essential for N4 replication, including DNA helicase (ORF50, N4 gp37-like), DNA polymerase (ORF44, N4 gp39-like), primase (ORF32, N4 gp43-like), and single-stranded DNA-binding protein (ORF30, N4 gp45-like) are conserved in the genome. The packaging system components, including the portal protein and the terminase large subunit, were recognized as ORF10 and ORF3, respectively. No lysis cluster could be identified, and only two proteins containing either a metallopeptidase domain (ORF51) or a glycoside hydrolase family protein (ORF69) may be involved in host lysis. In N4-like phages, different groups of lysis clusters with variable gene content have been described previously^40^, such as phage VBP47, and VBP32, which also do not contain any identified lysis cluster. Most lysis clusters of N4-like phages were located upstream of the portal protein but may arrange in different orientations. In the case of *Vibrio* phage VCO139, endolysin is located upstream of RNA polymerase^34^. The alternative arrangement of lysis clusters made their identification much more difficult.

**Figure 5:**
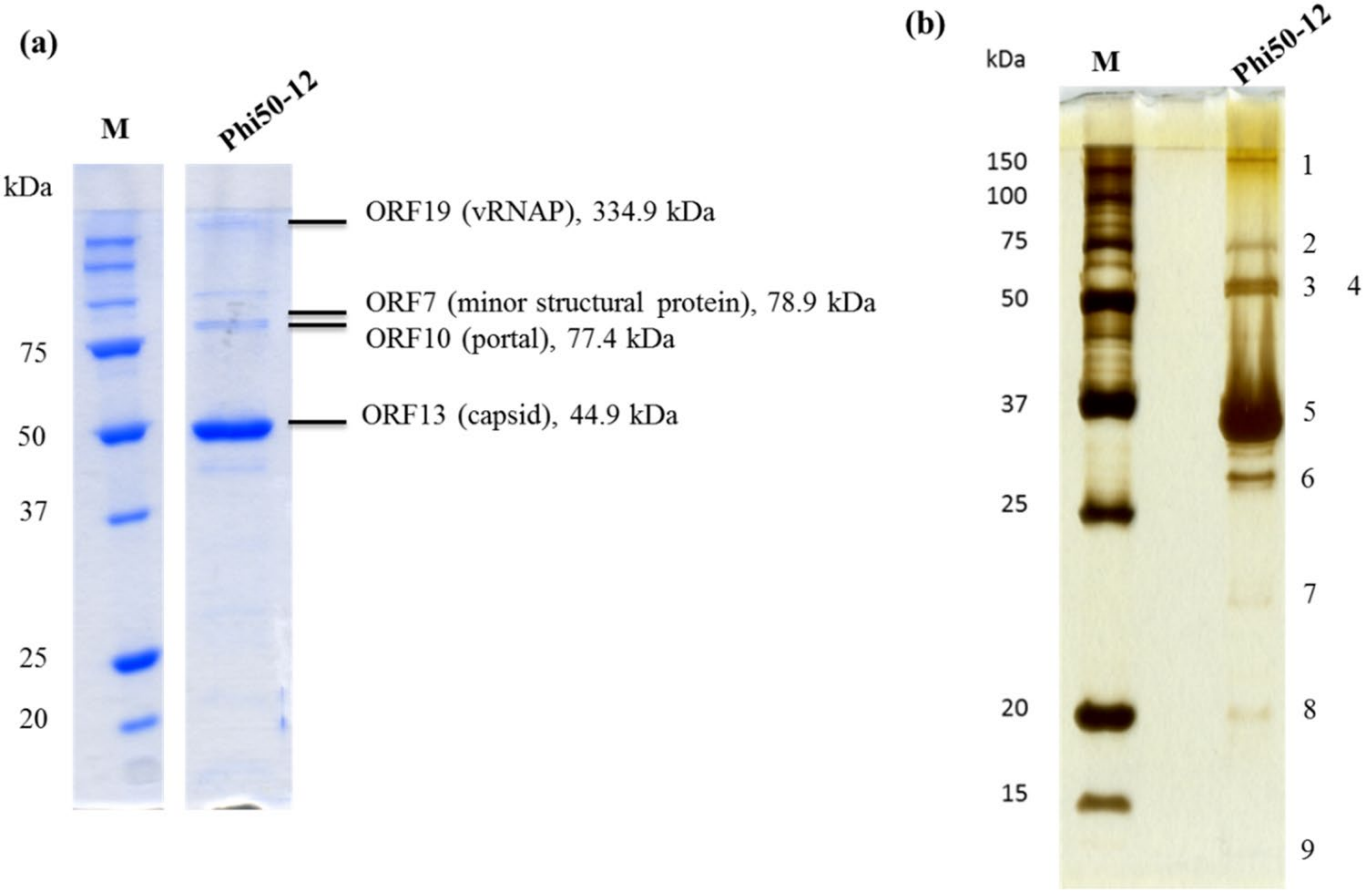
Structural protein analysis by 12% SDS-PAGE. (**a**) Phage particles (5 × 10^11^ PFU) were boiled in protein sample buffer (80 mM Tris, pH 6.8; 2% SDS; 2-mecraptoethanol; 0.0006% Bromophenol blue) and subjected to SDS-PAGE. Parallelly, the sliced bands were subjected to LC/MS/MS analysis. Peptides matched with the annotated ORFs, and their observed molecular mass were indicated. Four of them were identified to be virion-encapsuled RNA polymerase (ORF19), minor structural protein (ORF7), portal protein (ORF10) and capsid protein (ORF13), respectively. Original gel is presented in Supplementary Fig. S9. (**b**) For better visualization, a silver stain procedure was also performed, nine bands were visible on the gel. Numbers indicate the protein band location. M is the molecular weight marker, phi50-12 represents the structural protein lysate.

For better visualization and confirmation, we performed two staining methods to detect the structural proteins in phi50-12. The results showed that at least nine structural proteins of the phi50-12 virions were detected by SDS-PAGE (Fig. 5a,b). The gel band pattern is similar to the protein profiles of JA1 and VCO139, two closely related N4-like vibrio phages of *V. cholerae*^34^. The results revealed major bands corresponding to the major coat protein (ORF 13), vRNAP (ORF 19), minor structural proteins (ORF7), and portal protein (ORF10) (Fig. 5a,b). LC/MS/MS analysis was performed to confirm the identity of the structural proteins.

One minor structural protein (ORF5) was predicted to be a polysaccharide lyase enzyme due to its similarity with JA1_0065 in JA1. This enzyme is speculated to exhibit substrate specificity^34^.

### Comparative genomics

**Figure 6:**
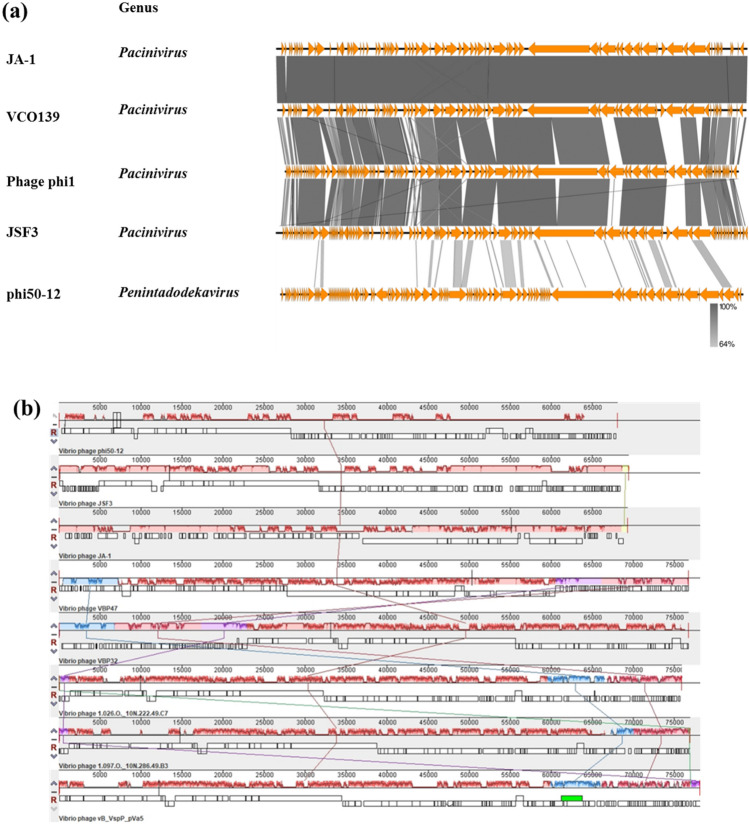
Comparative genomic analysis of N4-like *Vibrio* phages. (**a**) TBLASTX was performed by Easyfig. All the phages belong to N4-like Vibrio phages. “Genus” represents the taxonomy to which they belong. The similarity range is indicated by the gradient scale. (**b**) Mauve alignment of selected N4-like Vibrio phages infecting a variety of *Vibrio* hosts. The hosts include *V. owensii* (phi50-12), *V. cholera* (JSF3, JA-1), *V. parahaemolyticus* (VBP47, VBP32), unidentified hosts (*Vibrio* phage 1.026.O._10N222.49.C7, 1.097.O._10N.286.49.B3), *Vibrio splendidus* (vB_VspP_pVa5). Each colored block represents a region of collinear sequence (LCB, local collinear block) among genomes. White boxes represent the ORFs of each genome and their arrangements.

The nucleotide sequence of phi50-12 did not show any closely related sequences in the public database; however, the annotated ORFs matched some N4-like vibrio phages. Therefore, we performed genomic comparisons using Easyfig and Mauve alignment to demonstrate the conserved part of phi50-12 with N4-like vibrio phages. The results showed that the conserved part of the genomic organization of phi50-12 was located in the core region of genomes of N4-like vibrio phages (genome size smaller than 70 kb) (Fig. 6a) and short fragments of the collinear region of selected N4-like vibrio phages (Fig. 6b). Both results confirmed that phi50-12 is a novel *Vibrio* N4-like phage. All smaller genomes of N4-like vibrio phages, including phi50-12, lack a fragment encoding N4 gp24 and gp25 (rIIA- and rIIB-like proteins); therefore, their genome lengths are shorter than those of other N4-like phages or N4-like vibrio phages. rIIA and rIIB have been reported to be involved in preventing host lysis during phage propagation^41^; therefore, the burst sizes of N4 and *Achromobacter* phage phiAxp-3 are relatively large, reaching 3000–9000 PFU/infected cells^42,43^. In phi50-12, the burst size was 106 PFU/infected cells, much less than the N4 burst size, and electron micrography confirmed that no aggregations of virions were present inside the cell (Fig. 2b,c and Supplementary Fig. S3). Whether or not the lack of rIIA and rIIB fragments influences the burst size needs to be determined in further studies.

### Phylogenetic analysis

**Figure 7:**
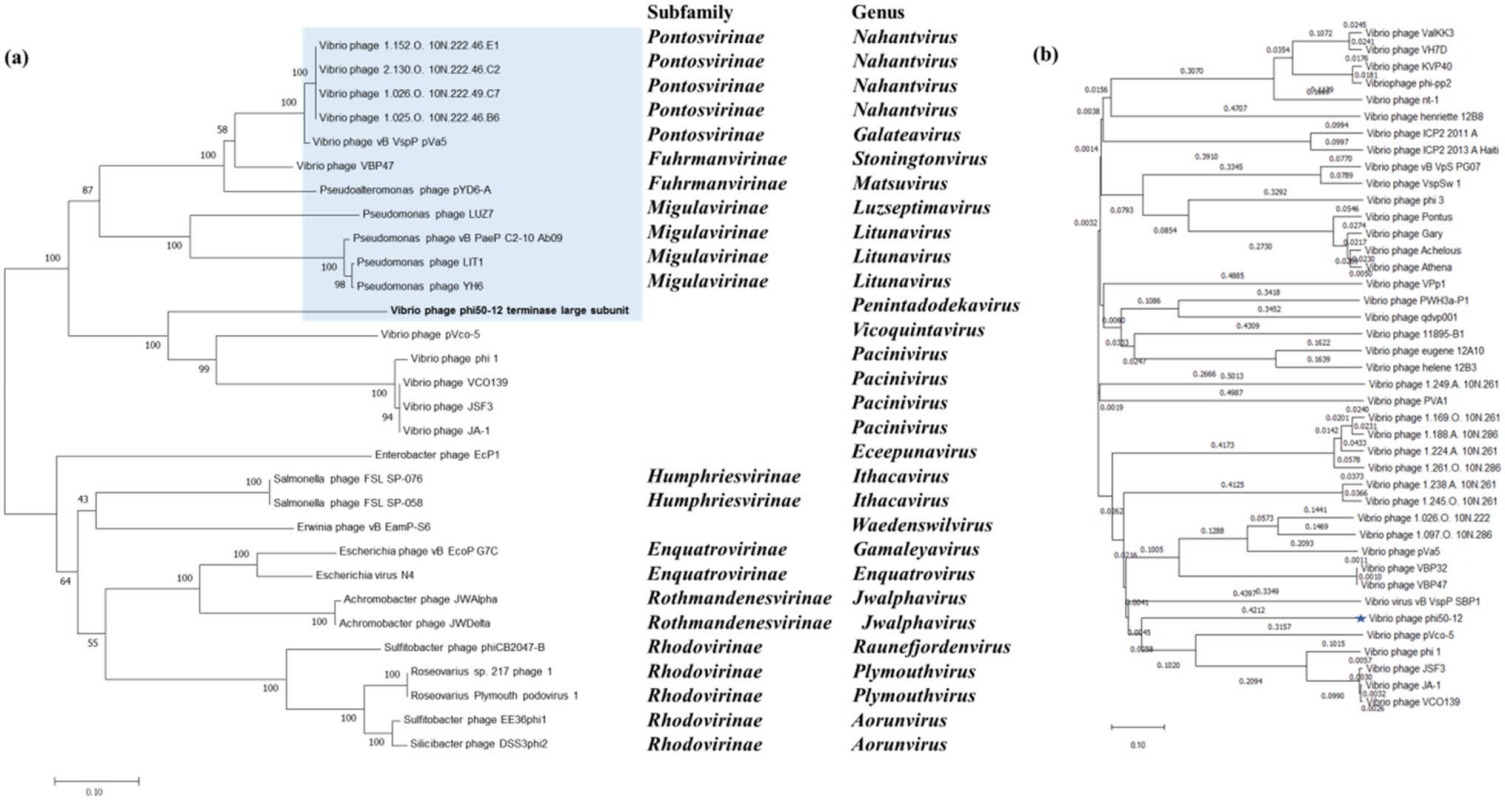
Phylogenetic analysis of terminase large subunit and viral proteomic tree analysis. (**a**) The tree generated by MEGA11 based on Neighbor-joining method with 1000 bootstraps. The subfamily and genus of the phages are indicated on the right. (**b**) Viral proteomic tree generated by VIPTree. This analysis involved all Vibrio phages available in the reference database at present.

Due to the increasing number of N4-like members, ICTV has accepted and reclassified the N4 phage and N4-like phages into a new family, *Schitoviridae*, named after Giancarlo Schito^44^. This family comprises 8 subfamilies and 22 new genera. To determine the phylogenetic relationship between phi50-12 and other N4-like phages, we chose terminase (Fig. 7a), vRNAP (Supplementary Fig. S8), and DNA polymerase (DNAP) (Supplementary Fig. S8) as candidates to collect the corresponding sequences from the available orthologous sequences of N4-like phages and performed phylogenetic analysis. The results demonstrated that phi50-12 was present as a single branch, which now has been placed under genus *Penintadodekavirus* as species *penintadodekavirus* 5012, but shared the same group as smaller N4-like vibrio phages. The most closely related genome was that of pVco5, a phage that infects *Vibrio coralliilyticus* and belongs to a new genus *Vicoquintavirus*. All three genes showed a similar tree patterns. We also performed a genome-wide comparison with all vibrio phages using VIPTree^45^. The proteomic tree demonstrated similar results in a single gene tree (Fig. 7b); phi50-12 clustered with N4-like vibrio phages and formed a single branch, but was close to the genomes of N4 vibrio phages. This may indicate that the intertidal zone is a specific microenvironment for the evolution of the unique features of phi50-12.

### Assessment of bacterial growth inhibition of phi50-12 in zebrafish

**Figure 8:**
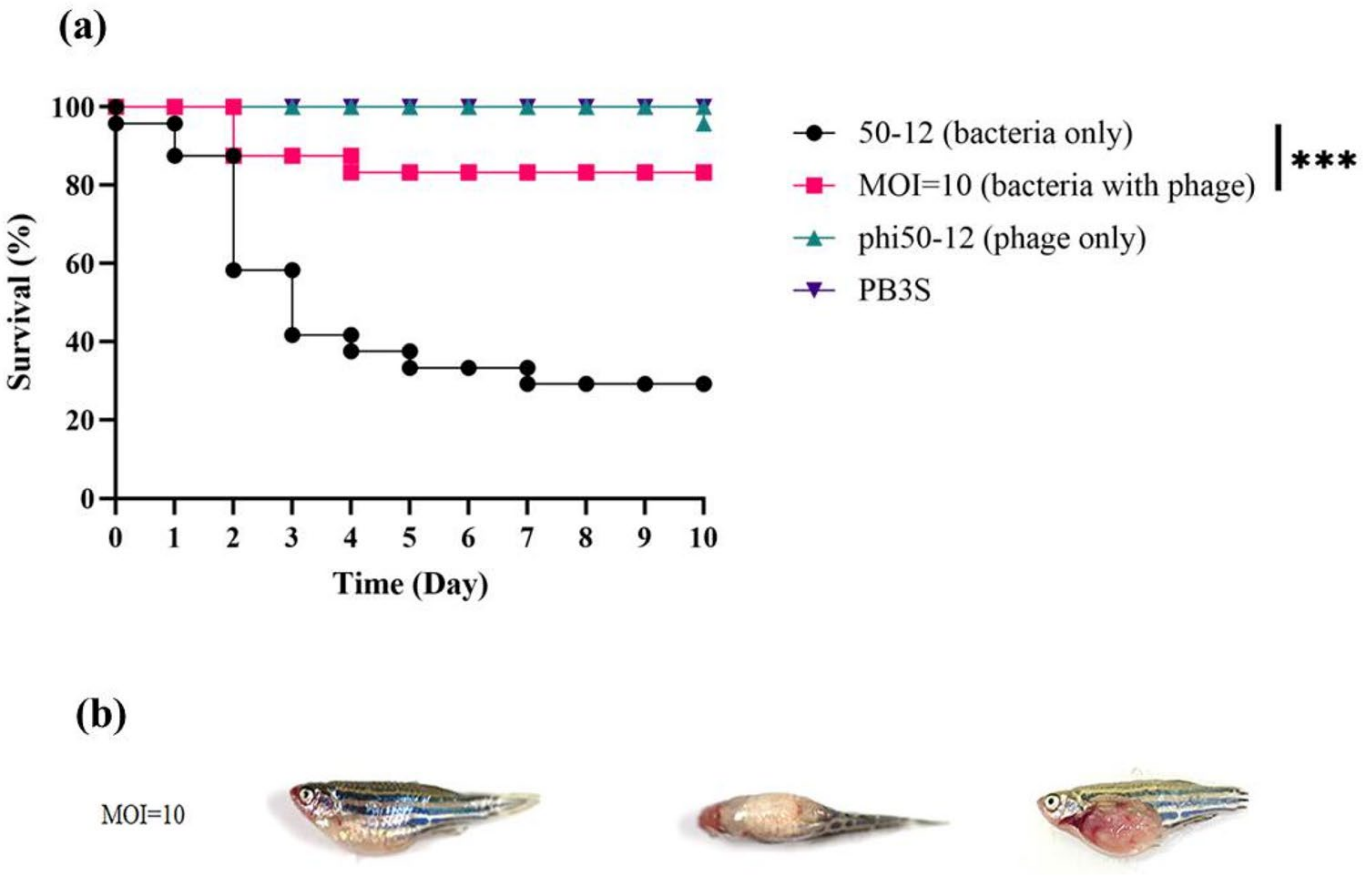
Therapeutic effect of phi50-12 in zebrafish. (**a**) Four groups were considered, each containing 24 fishes. These were subjected to four distinct conditions: injection with *V. owensii* GRA50-12 cells (2.8–4.5 × 10^6^ CFU/20 µl, black line), phi50-12 treatment (MOI = 10) for 30 min followed by challenge with bacteria (red line), phi50-12 treatment only (green line) and PB3S buffer as negative control (purple line); and survival rates were measured. The X-axis represents the time post-infection and the Y-axis represents the survival percentage of the zebra fish. (**b**) The disease symptoms of zebrafish rescued by phi50-12 with MOI = 10. *V. owensii* GRA50-12 introduced first in the fish followed by administration of phi50-12 after 30 min. MOI = 10 represents the disease symptom of sick fish in the group with phi50-12 administration. Left: side view; middle: top view; right: abdomen anatomy. The anatomical picture showed swollen abdomen and bleeding intestines. The significance of the differences between groups was performed by Log-rank and Gehan–Breslow–Wilcoxon test in GraphPad Prism 9. “***” indicates p < 0.001.

The application of phage therapy instead of antibiotics to eliminate pathogenic bacteria in *Vibrio* to reduce fish mortality in aquaculture has been previously reported^46–48^. Thus, the reduced bacterial growth by phi50-12 increased the survival of zebrafish was measured using the cumulative mortality over 10 days. The efficacy to reduce the mortality of fish with phi50-12 was measured after challenge with GRA50-12 for 30 min, followed by injection with phi50-12 at MOI = 10. Compared to the GRA50-12 challenged group, phi50-12 administration significantly reduced cumulative mortality within 3 days (41.6% vs. 87.5%). After 10 days, only four zebrafish died when treated with phi50-12, whereas 17 died in the GRA50-12 challenged group. Fish injected with phi50-12, or with PB3S buffer only, remained alive until the end of the experiment (Fig. 8a). The disease symptoms of GRA50-12 infected zebrafish were monitored; whereas PB3S buffer and phi50-12 injected groups showed no disease symptoms (Fig. 8b, Control/PB3S). The zebrafish injected with GRA50-12 in the absence or presence of phi50-12 showed a swollen abdomen and bleeding on the body surface (Figs. 1b and 8b, MOI = 10), but the symptoms were mild in the phi50-12 rescued group. This suggests that phi50-12 administration can cease the symptoms of diseased fish successfully, in most cases. The anatomy of diseased fish in the phi50-12 rescued group also showed swollen and bleeding intestines. One fish died on the 10th day in the phi50-12 only group, which was assumed to be a natural death.

## Conclusions

This study identified the coral symbiont *V. owensii* GRA50-12 as a potent pathogen using phenotypical, biochemical, and zebrafish toxicity tests. Many *Vibrio* species in the Harveyi clade are pathogenic to aquatic animals, especially to shrimp larvae, lobster, juvenile fish, shellfish, mollusks^49^ and corals^50^. This has negatively affected the economy of the aquaculture industry worldwide. A virulent phage belonging to the species *Schitoviridae* of the N4-like phage family^44^ was isolated and characterized. The sequence showed novelty with no closely related sequences. Although, phi50-12 has a limited host range, it showed sensitivity to *Vibrio *spp. isolated from the same ecological niche. Phi50-12 has a short adsorption time, a large burst size, and stability over a wide range of pH and temperatures. In addition, in vitro and in vivo analyses showed that phi50-12 had a strong lytic ability against *V. owensii* GRA50-12. Complete genome and phylogenetic analysis of phi50-12 confirmed the absence of any antibiotic resistance genes or virulence factors. This study is the first to report the pathogenicity of localized *V. owensii* in Taiwan and its interaction with a newly isolated phage. The measurement of survival in the zebrafish model suggests that phi50-12 is a good candidate for reducing the population of *V. owensii*, which may be useful for the prevention and control of diseases caused by *V. owensii.* However, extensive studies must be carried out further so as to ascertain these therapeutic effects.

## Materials and methods

### Conditions of cultivation

All *Vibrio* spp. used in this study are listed in Table 1, and most of them (except those where the sources are specified) were originally isolated from several kinds of macroalgae on corals in the intertidal zone of Shihti Fishing Port in the east of Hualien County. The water samples were provided by Dr. Chen CY (Department of Life Science, Tzu Chi University). Cultures were grown at 30 °C in TSB medium (BD-Difco, USA) with 3% of NaCl and aerated by shaking at 150 rpm. TSB3S containing 1.5% agar was used for routine plate cultures, whereas TSB3S containing 0.75% agar for the double agar overlay method was used for the spot test and titration of the phage in the plaque assay.

### Phage isolation and purification

Phage phi50-12 was isolated from a water sample from the intertidal zone around the location where the host *V. owensii* GRA50-12 was isolated. The collected water sample was filtered through a 0.22 µm filter membrane. The filtered water sample (9.0 ml) was mixed with 50 μl of *V. owensii* GRA50-12 cells grown to log phase (2 × 10^8^ CFU/ml). The mixtures were grown overnight to enrich for possible existing phages. Furthermore, 5 μl of the culture supernatant was spotted onto the bacterial lawns grown on soft agar (0.75%) using the double agar overlay method. The clearing zones formed on the lawns were subjected to three rounds of single plaque isolation. A single plaque on the lawn of GRA50-12 was picked and enriched thrice to obtain a pure phage population. High titer lysates of the phage (50 ml containing ~ 10^12^ PFU/ml) were concentrated, centrifuged at 46,500*g* (Beckman Coulter Avanti centrifuge, J25I rotor; Beckman Coulter, Brea, CA, USA) for 3 h at 4 °C. The pellets were suspended in 1.0 ml SM buffer (0.05 M Tris–HCl, pH 7.5, 0.1 M NaCl, 0.008 M MgSO_4_·7H_2_O, and 0.01% gelatin) and loaded onto block gradients of CsCl (1.3, 1.4, 1.45, 1.5, 1.6 and 1.7 g/ml), followed by ultracentrifugation (154,000*g* for 3 h at 4 °C with an SW41Ti rotor in an Optima LE-80K Ultracentrifuge from Beckman Coulter). The phage particles were recovered from the gradient and desalted by dialysis.

### Phage DNA isolation and pulsed-field gel electrophoresis

We followed the procedures described by Chang et al.^51^ to isolate phage DNA and perform restriction enzyme digestion. PFGE was performed as previously described^52^, and the CHEF-DR III System (Bio-Rad Laboratories, Hercules, CA, USA) was used under the following conditions: 9 °C in 0.5× Tris–borate–EDTA buffer (pH 8.0) at 6 V/cm with pulse ramps from 3.5 to 4 s for 19.5 h. A Midrange IPFG Marker (New England Biolabs, Ipswich, MA, USA) was used as the molecular size standard.

### Genome analysis

The genomic sequence of phi50-12 was determined by the Genomics Company, Taiwan. The average genome coverage is (approximately) 2729×.

Genome annotation was performed using Glimmer^53^ and GeneMark^54^. We also referred to InteroProScan (https://www.ebi.ac.uk/interpro/search/sequence/) and HHpred (https://toolkit.tuebingen.mpg.de/tools/hhpred) as tools to obtain best possible annotation for phage phi50-12. Functional predictions were made using BLASTP. Potential tRNA and tmRNA genes were predicted by Aragorn et al.^35^ and tRNAScanSE^36^. Antibiotic genes and virulence factors were identified using ResFinder and VirulenceFinder^36^. Phylogenetic analysis was performed using MEGA 11 software^55^. Comparative genomic analysis was performed using Easyfig 2.2.3^56^ and Mauve alignment^57^.

### Transmission electron microscopy

Purified phage particles were applied to the surface of a formvar-coated grid, negatively stained with 1% phosphotungstic acid (PTA), and then examined by TEM (Hitachi Company, Japan).

### Determination of the phage adsorption and growth curve

Phage adsorption curve was determined by growing host cells to log phase (~ 2 × 10^8^/ml after 1.5 h subculturing), then infected with phi50-12 at an MOI of 0.0005, and incubated at 30 °C. Aliquots of 500 μl were taken at an interval of every 3 min and centrifuged (12,000×*g*, 5 min). 100 μl of supernatant was removed to calculate the titer of unadsorbed phages. One-step growth and burst-size measurements were performed as previously reported^58^. All experiments were performed at least twice in triplicates.

### Stability to physical, chemical and environmental factors

Except for UV radiation treatment (10^3^ PFU/ml), a phage suspension of concentration 4 × 10^6^ PFU/ml was used for all individual treatments. For temperature sensitivity tests, phage suspensions were incubated at different temperatures for 24 h. Salinity tests were carried out by incubating phage suspensions in different percentages of NaCl-containing media for 24 h. To simulate phage exposure under UV radiation in a natural environment, phage suspensions were diluted to 10^3^ PFU/ml and exposed to UV-C radiation for 15 min and 24 h. The loss of infectivity was measured using the plaque-forming assay method.

### Structural proteins analysis

The phage particles were purified using CsCl and concentrated. The suspension was mixed with protein sample buffer, boiled for 10 min, and then separated on a 12% SDS-PAGE gel. Protein size markers were purchased from Bio-Rad, and the protein bands were visualized using InstantBlue (Expedeon Protein Solutions, Ltd., Cambridge, UK). For better visualization of low-copy structural proteins, gels were stained using the silver staining method^59^. The identity of the virion proteins was elucidated using LC/MS/MS (Energenesis Biomedical Co., Ltd., Taiwan).

### In vitro lysis

Mid-log phase cells were loaded into 96-well microplate and mixed with different MOIs of phi50-12 in 200 μl medium in triplicate. The plates were incubated at 30 °C. At each time point, the optical density of the plate was measured at a wavelength of 600 nm (Thermo Scientific Varioskan^®^ Flash). Three wells that were not loaded with phi50-12 served as the controls. The cell lysis curve was monitored every 30 min for 10 h.

### Host range analysis

The host range was determined by spotting 5 µl of phage lysate on the lawns of investigated cultures on TSB3S agar plates, as previously described^58^. A clear spot indicates host sensitivity.

### Zebrafish infections

The zebrafish (*Danio rerio*) lines used in this study were the wild type AB variety which were maintained and grown in the Tzu Chi University FishCore facility according to standard protocols. All protocols in accordance with the guidelines and regulations of council of Agriculture Executive Yuan (Taiwan) for the care and use of laboratory animals. Mixed male and female populations of zebrafish were kept in 9 L tanks at 28 °C and maintained in a 14 h light/10 h dark cycle. All methods used in this study were performed in accordance with the guidelines and regulations of Institutional Animal Care and Use Committee (IACUC) in Tzu-Chi University.

For confirmation of pathogenicity of GRA50-12, a total of 24 adult male and female fish (approximately 3–3.5 cm) were mixed and divided into three groups (8 fishes/ group). Two different bacterial cell counts (2.2 × 10^6^ CFU/20 µl and 2.2 × 10^4^ CFU/20 µl) were applied and one used PB3S as control. Before injection, individuals anesthetized with 160 mg/ml tricaine then were injected intraperitoneally with insulin needle.

For the measurement of reduced bacterial growth by phi50-12, mixed adult male and female fish were grouped into 24 fishes per group. One group was injected with 20 µl (2.8–4.5 × 10^7^ CFU) of *V. owensii* GRA50-12 suspended in PB3S through the cloaca with an insulin needle. The other group was infected with the same dose of *V. owensii* GRA50-12 and injected 30 min later with a dose of phi50-12 (MOI = 10 in 20 µl) into the cloaca. phi50-12 treatment only and PB3S buffer as negative control. The significance of the differences between groups was determined using the Log-rank and Gehan–Breslow–Wilcoxon test in GraphPad Prism 9.

Zebrafish use was performed in agreement with ARRIVE guidelines (https://arriveguidelines.org). The laboratory protocol was approved by the Institutional Animal Care and Use Committee of Tzu Chi University (IACUC approval no. 109018).

### Nucleotide sequence accession number

The genome sequence of phi50-12 was deposited in GenBank under accession number MN584918.1.

### Institutional Review Board statement

The laboratory protocol was approved by the Institutional Animal Care and Use Committee of Tzu Chi University (IACUC Approval No.: 109018).

## Supplementary Information

Supplementary Information.

## Data Availability

The data presented in this study are available in this article and supplementary material here.
