# Supplementary material for: Isolation and characterization of a Vibrio owensii phage phi50-12
Source: Sci Rep. 2022 Sep 30;12:16390. doi: 10.1038/s41598-022-20831-2 (PMC9525291; doi:10.1038/s41598-022-20831-2)
Supplement: Supplementary file 1 — Supplementary Information. [file 41598_2022_20831_MOESM1_ESM.pdf]

Supplementary Table S1: ***Vibrio owensii*** **(GCA_000829735.1) genome neighbors**

https://www.ncbi.nlm.nih.gov/genome/neighbors/17721?genome_assembly_id=218358

#Organism/Name	Strain	BioSample	BioProject	Assembly	Level	Size	Scaffolds	Symmetric Identity	Gapped Identity
Vibrio owensii	OCN002	SAMD00019142	PRJDB3039	GCA_000818275.1	Contig	5.85079	198	90.0948	97.0249
Vibrio owensii	bablab_jr007	SAMN15545057	PRJNA646503	GCA_016464175.1	Contig	5.85456	145	89.882	96.7716
Vibrio owensii	00-18-3-1	SAMN12668384	PRJNA563078	GCA_013114245.1	Contig	5.81745	55	89.7277	97.056
Vibrio owensii	XSBZ03	SAMN05213947	PRJNA314425	GCA_002021755.1	Complete Genome	5.8902	-	89.5139	97.0746
Vibrio owensii	BR345	SAMN11039182	PRJNA524972	GCA_004349325.1	Contig	5.81242	38	89.3944	97.0474
Vibrio owensii	bablab_jr005	SAMN15545055	PRJNA646503	GCA_016464195.1	Contig	5.8597	142	89.3292	96.7482
Vibrio owensii	NCCB 76037	SAMN04279320	PRJNA302718	GCA_001557965.1	Contig	5.80893	54	88.3445	96.9731
Vibrio owensii ATCC 25919	ATCC 25919	SAMD00036653	PRJDB720	GCA_000400225.1	Scaffold	5.80452	174	88.3036	96.9748
Vibrio owensii	NCTC11318	SAMEA1041671	PRJEB6403	GCA_90046039	Contig	5.8642	7	88.0961	96.978

		99		5.1		9			5
**Vibrio owensii**	**051011B**	**SAMD00019129**	**PRJNA429421**	**GCA_002887655.1**	**Complete Genome**	**6.26138**	**-**	**86.9108**	**97.0643**
**Vibrio owensii**	**051011B**	**SAMD00019129**	**PRJDB3039**	**GCA_000829635.1**	**Scaffold**	**6.06628**	**133**	**86.8501**	**97.0881**
**Vibrio owensii**	**47666-1**	**SAMD00019139**	**PRJDB3039**	**GCA_000818255.1**	**Contig**	**5.87115**	**233**	**86.849**	**96.7538**
**Vibrio owensii 47666-1**	**47666-1**	**SAMN02946480**	**PRJNA207882**	**GCA_000817825.1**	**Contig**	**5.83372**	**55**	**86.7738**	**96.7529**
**Vibrio owensii**	**bablab_jr002**	**SAMN15545052**	**PRJNA646503**	**GCA_016464265.1**	**Contig**	**6.26993**	**250**	**86.6419**	**96.7386**
**Vibrio owensii LMG 25430**	**LMG 25430**	**SAMD00036658**	**PRJDB725**	**GCA_000400325.1**	**Scaffold**	**5.82496**	**341**	**86.4464**	**96.6788**
**Vibrio owensii**	**V180403**	**SAMN09878769**	**PRJNA487170**	**GCA_003691545.1**	**Complete Genome**	**6.41856**	**-**	**86.3545**	**97.0816**
**Vibrio owensii**	**SH14**	**SAMN04128477**	**PRJNA297683**	**GCA_001310575.2**	**Complete Genome**	**6.26821**	**-**	**85.1883**	**96.995**
**Vibrio owensii**	**bablab_jr009**	**SAMN15545059**	**PRJNA646503**	**GCA_016464115.1**	**Contig**	**6.19774**	**207**	**85.1873**	**96.8454**

**Vibrio owensii**	**1700302**	**SAMN09874178**	**PRJNA487143**	**GCA_003691505.1**	**Complete Genome**	**6.42431**	**-**	**84.1206**	**97.0009**
**Vibrio owensii CAIM 1854 = LMG 25443**	**DY05**	**SAMN02946409**	**PRJNA189697**	**GCA_000817815.1**	**Scaffold**	**6.34538**	**119**	**80.7687**	**96.8336**
**Vibrio owensii CAIM 1854 = LMG 25443**	**CAIM 1854 = LMG 25443**	**SAMD00036661**	**PRJDB728**	**GCA_000400385.1**	**Contig**	**6.36686**	**451**	**80.7192**	**96.8439**
**Vibrio owensii**	**20160513VC2W**	**SAMN09536720**	**PRJNA479398**	**GCA_013459835.1**	**Complete Genome**	**7.25204**	**-**	**78.7005**	**97.0351**
**Vibrio owensii**	**ISF-69-8**	**SAMN28767316**	**PRJNA843723**	**GCA_024746155.1**	**Contig**	**6.03024**	**67**	**-**	**-**

Supplementary Table S2 Biochemical anaysis of *Vibrio owensii* GRA50-12 using API20E.

TESTS	SUBSTRATE	REACTION TESTED	-RESULTS	+RESULTS
ONPG	ONPG	Beta-galactosidase	Colorless	Yellow (+)
ADH	Arginine	Arginine dihydrolase	Yellow (-)	Red/orange
LDC	Lysine	Lysine decarboxylase	Yellow	Red/orange (+)
ODC	Ornithine	Ornithine decarboxylase	Yellow	Red/orange (+)
CIT	Citrate	Citrate ultilization	Pale (-) green/yellow	Blue-green/blue
H2S	Na thiosulfate	H2S production	Colorless/grey (-)	Black deposit
URE	Urea	Urea hydrolysis	Yellow (-)	Red/orange
TDA	Tryptophan	Deaminase	Yellow(-)	Brown-red
IND	Tryptophan	Indole production	Yellow	Rdn(2min.) (+)
VP	Na pyruvate	Acetoin production	Colorless (-)	Pink/red(10 min.)
GEL	Charcoal gelatin	Gelatinase	No diffusion of black	Black diffuse (+)
GLU	Glucose	Fermentation/oxidation	Blue/blue-green	Yellow (+)

**MAN**	**Mannitol**	**Fermentation/oxidation**	**Blue/blue-green**	**Yellow (+)**
**INO**	**Inositol**	**Fermentation/oxidation**	**Blue/blue-green (-)**	**Yellow**
**SOR**	**Sorbitol**	**Fermentation/oxidation**	**Blue/blue-green (-)**	**Yellow**
**RHA**	**Rhamnose**	**Fermentation/oxidation**	**Blue/blue-green (-)**	**Yellow**
**SAC**	**Sucrose**	**Fermentation/oxidation**	**Blue/blue-green**	**Yellow (+)**
**MEL**	**Melibiose**	**Fermentation/oxidation**	**Blue/blue-green (-)**	**Yellow**
**AMY**	**Amygdalin**	**Fermentation/oxidation**	**Blue/blue-green**	**Yellow (+)**
**ARA**	**Arabionse**	**Fermentation/oxidation**	**Blue/blue-green (-)**	**Yellow**
**OX**	**oxidase**	**oxidase**	**Colorless/yellow**	**Violet (+)**

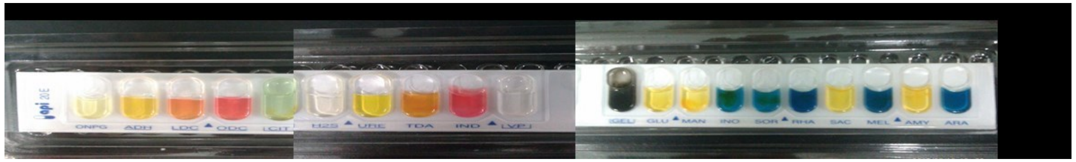

The result of A-/L+/O+ represents arginine dihydrolase (A)-negative, lysine decarboxylase (L)-positive, and ornithine decarboxylase (O)-positive. It is a typical reaction for bacteria in the Harveyi clade, which do not use citrate as a carbon source, but are able to produce acid from amygdalin.

Supplementary Table S3. Functional annotation of the genome of phi50-12

orf	Start	Stop	Length (aa)	Identity	Accession	Related protein	Protein product
01	329	631	100	87/97(90%)	YP_009009731.1	hypothetical protein (Vibrio phage PVA1)	hypothetical protein
02	747	1421	224	100/230(43%)	YP_010089861.1	hypothetical protein (Vibrio phage pVco-5)	hypothetical protein
03	1414	2988	524	334/523(64%)	YP_010089985.1	terminase large subunit (Vibrio phage pVco-5)	terminase large subunit
04	2996	3637	213	98/228(43%)	YP_009874368.1	hypothetical protein (Vibrio phage VCO139)	hypothetical protein
05	3634	6426	930	261/627(42%)	WP_142740706.1	hypothetical protein (*Vibrio cholerae*)	hypothetical protein/pectin lyase fold
06	6426	6599	57	-		No similarity	hypothetical protein
07	6690	8849	719	281/503(56%)	AUS02987.1	hypothetical protein (Vibrio phage	hypothetical protein/Phage T7

						2.275.O._10N.286.54.E11)	tail fiber protein; pfam03906
08	8861	9109	82	38/80(48%)	WP_102351169.1	hypothetical protein (*Vibrio cyclitrophicus*)	hypothetical protein
09	9143	9526	127	-		No similarity	hypothetical protein
10	9604	11658	684	338/689(49%)	YP_009874364.1	portal protein (Vibrio phage VCO139)	portal protein
11	11664	12005	113	-		No similarity	hypothetical protein
12	12012	13043	363	100/287(35%)	DAE92006.1	TPA: MAG TPA: hypothetical protein (*Podoviridae* sp. ctXdu7)	hypothetical protein
13	13054	14286	410	212/383(55%)	WP_126055512.1	N4-gp56 family major capsid protein (*Mesorhizobium* sp. M4B.F.Ca.ET.058.02.1.1)	major capsid protein
14	14298	14945	215	58/117(50%)	MBP93671.1	hypothetical protein (Flavobacteriaceae bacterium)	hypothetical protein
15	14945	15454	169	56/142(39%)	YP_009198596.1	hypothetical protein (Vibrio phage phi 1)	hypothetical protein

16	15524	17569	681	242/709(34%)	YP_008126819.1	hypothetical protein (Vibrio phage JA-1)	hypothetical protein/N4 gp53
17	17556	17978	140	36/64(56%)	WP_102396907.1	hypothetical protein (*Enterovibrio norvegicus*)	hypothetical protein/N4 gp52
18	17980	19173	397	67/210(32%)	YP_010089973.1	hypothetical protein (Vibrio phage pVco-5)	hypothetical protein
19	19185	28259	3024	934/3126(30%)	YP_009874355.1	virion-encapsulated RNA polymerase (Vibrio phage VCO139)	virion-encapsulated RNA polymerase/N4 gp50
20	28281	28601	106	-		No similarity	hypothetical protein
21	28594	28914	106	-		No similarity	hypothetical protein
22	28915	29253	112	-		No similarity	hypothetical protein
23	29264	29455	63	-		No similarity	hypothetical protein
24	29452	29667	71	48/65(74%)	QMP81715.1	hypothetical protein (Vibrio phage XM1)	hypothetical protein

25	29668	29841	57	-		No similarity	hypothetical protein
26	29903	30436	199	-		No similarity	hypothetical protein
27	30429	30752	107	35/96(36%)	NIV12308.1	hypothetical protein (*Aliifodinibius* sp.)	hypothetical protein
28	30770	31201	143	86/139(62%)	WP_034876896.1	hypothetical protein (*Endozoicomonas montiporae*)	hypothetical protein
29	31204	31587	127	88/125(70%)	MBR9878665.1	Holliday junction endonuclease (Gammaproteobacteria bacterium)	hypothetical protein/ Holliday junction endonuclease
30	31662	32384	240	78/247(32%)	RLC98789.1	hypothetical protein (Chloroflexi bacterium)	hypothetical protein
31	32442	33194	250	124/250(50%)	YP_009198587.1	AAA domain protein (Vibrio phage phi 1)	hypothetical protein/AAA domain protein
32	33237	35411	74	397/719(55%)	YP_010089961.1	hypothetical protein (Vibrio phage pVco-5)	hypothetical protein/ N4 gp43/ primase

33	35411	35641	76	-		No similarity	hypothetical protein
34	35648	36655	335	161/335(48%)	YP_010089960.1	hypothetical protein (Vibrio phage pVco-5)	hypothetical protein/ N4 gp42
35	36808	37323	171	61/162(38%)	YP_009914108.1	hypothetical protein (*Pseudomonas* phage Epa13)	hypothetical protein
36	37434	38231	265	59/186(32%)	MBL4909043.1	ParB N-terminal domain-containing protein (Alteromonadaceae bacterium)	hypothetical protein
37	38222	38827	201	117/196(60%)	MBL4623368.1	hypothetical protein (Immundisolibacteraceae bacterium)	hypothetical protein
38	38834	39166	110	-		No similarity	hypothetical protein
39	39163	39360	65	29/64(45%)	YP_010093499.1	hypothetical protein (Vibrio phage 1.245.O._10N.261.54.C7)	hypothetical protein
40	39364	39609	81	29/79(37%)	WP_045035730.1	hypothetical protein (*Photobacterium iliopiscarium*)	hypothetical protein

41	39732	39992	86	-		No similarity	hypothetical protein
42	40004	40252	82	-		No similarity	hypothetical protein
43	40256	40615	119	49/109(45%)	APC44391.1	hypothetical protein (*Pseudoalteromonas* phage PH357)	hypothetical protein
44	40619	43243	874	446/841(53%)	YP_010089956.1	DNA polymerase (Vibrio phage pVco-5)	DNA polymerase
45	43243	43503	86	-		No similarity	hypothetical protein
46	43496	43750	84	-		No similarity	hypothetical protein
47	43743	44000	85	-		No similarity	hypothetical protein
48	43993	44448	151	-		No similarity	hypothetical protein
49	44429	44680	83	-		No similarity	hypothetical protein
50	44677	45837	386	121/392(31%)	YP_010089954.1	hypothetical protein (Vibrio phage pVco-5)	hypothetical protein
51	45932	47137	401	153/409(37%)	YP_008126799.1	metallopeptidase domain protein (Vibrio phage	hypothetical protein/

						JA-1)	metallopeptidase domain protein
52	47185	48354	389	109/273(40%)	YP_009198571.1	hypothetical protein (Vibrio phage phi 1)	hypothetical protein
53	48416	48820	134	46/118(39%)	YP_010089942.1	hypothetical protein (Vibrio phage pVco-5)	hypothetical protein
54	48834	49469	211	76/124(61%)	SCM71617.1	conserved hypothetical protein (uncultured *Pleomorphomonas* sp.)	hypothetical protein
55	49466	49630	54	-		No similarity	hypothetical protein
56	49630	49917	95	49/93(53%)	AUR89987.1	hypothetical protein (Vibrio phage 1.135.O._10N.222.54.B6)	hypothetical protein
57	49914	50267	117	-		No similarity	hypothetical protein
58	50274	51086	270	164/268(61%)	YP_008239443.1	hypothetical protein (*Salmonella* phage FSL SP-058)	hypothetical protein
59	51083	51667	194	108/185(58%)	QUE30147.1	hypothetical protein (Vibrio phage vB_ValP_IME234)	hypothetical protein

60	51679	51924	81	-		No similarity	hypothetical protein
61	52070	52402	110	-		No similarity	hypothetical protein
62	52405	54075	556	-		No similarity	hypothetical protein
63	54114	54341	75	-		No similarity	hypothetical protein
64	54345	54692	115	21/47(45%)	MBO5293219.1	transcription repressor NadR (Lachnospiraceae bacterium)	hypothetical protein
65	55019	55681	220	48/100(48%)	WP_180912452.1	MULTISPECIES: hypothetical protein (*Vibrio harveyi* group)	hypothetical protein
66	55732	55974	80	33/68(49%)	WP_059010108.1	hypothetical protein (Type-C symbiont of *Plautia stali*)	hypothetical protein
67	56140	56268	42	-		No similarity	hypothetical protein
68	56284	56823	179	-		No similarity	hypothetical protein
69	56862	57353	163	81/147(55%)	WP_112404091.1	glycoside hydrolase family protein (*Vibrio*	baseplate central spike complex

						diazotrophicus)	protein
70	57357	57743	128	60/127(47%)	CAG22427.1	hypothetical protein (*Photobacterium profundum* SS9)	hypothetical protein
71	57736	58032	98	-		No similarity	hypothetical protein
72	58004	58303	99	-		No similarity	hypothetical protein
73	58290	58514	74	-		No similarity	hypothetical protein
74	58501	58761	86	-		No similarity	hypothetical protein
75	58733	58999	88	-		No similarity	hypothetical protein
76	58962	59255	97	-		No similarity	hypothetical protein
77	59248	59526	92	66/91(73%)	MBP7662271.1	hypothetical protein (*Shewanella* sp.)	hypothetical protein
78	59513	59755	80	-		No similarity	hypothetical protein
79	59752	59985	77	-		No similarity	hypothetical protein

80	59988	60197	69	-		No similarity	hypothetical protein
81	60197	60430	77	-		No similarity	hypothetical protein
82	60430	60666	78	-		No similarity	hypothetical protein
83	60666	60881	71	-		No similarity	hypothetical protein
84	60949	62160	403	173/407(43%)	YP_009874314.1	DNA-directed RNA polymerase RNAP2 (Vibrio phage VCO139)	DNA-directed RNA polymerase
85	62182	62376	64	-		No similarity	hypothetical protein
86	62369	62566	65	-		No similarity	hypothetical protein
87	62550	62768	72	-		No similarity	hypothetical protein
88	62761	62955	64	-		No similarity	hypothetical protein
89	62952	63920	322	105/311(34%)	YP_009876321.1	RNA polymerase (Vibrio phage JSF3)	DNA-directed RNA polymerase
90	63990	64226	78	-		No similarity	hypothetical protein

91	64238	64555	105	-		No similarity	hypothetical protein
92	64579	64839	86	-		No similarity	hypothetical protein
93	64936	65157	73	-		No similarity	hypothetical protein
94	65278	65556	92	-		No similarity	hypothetical protein
95	65589	65954	121	54/126(43%)	AOQ26695.1	hypothetical protein (Vibrio phage 2E1)	hypothetical protein/PadR family transcriptional regulator
96	65947	66162	71	-		No similarity	hypothetical protein
97	66228	66389	56	-		No similarity	hypothetical protein
98	66393	66854	156	57/126(45%)	AUR89196.1	hypothetical protein (Vibrio phage 1.121.O._10N.286.46.C4)	hypothetical protein
99	66858	67079	73	-		No similarity	hypothetical protein

100	67093	67293	66	-	No similarity	hypothetical protein
101	67606	67848	80	-	No similarity	hypothetical protein

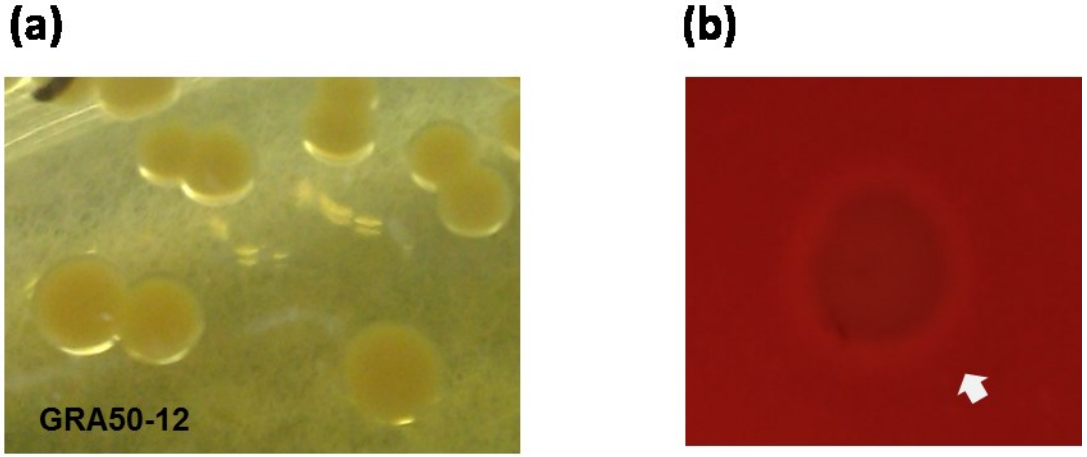

Supplementary Figure S1. Colony morphology and hemolytic activity of *V. owensii* GRA50-12 in **(a)** TSB3S and **(b)** Wagatsuma blood agar plate.

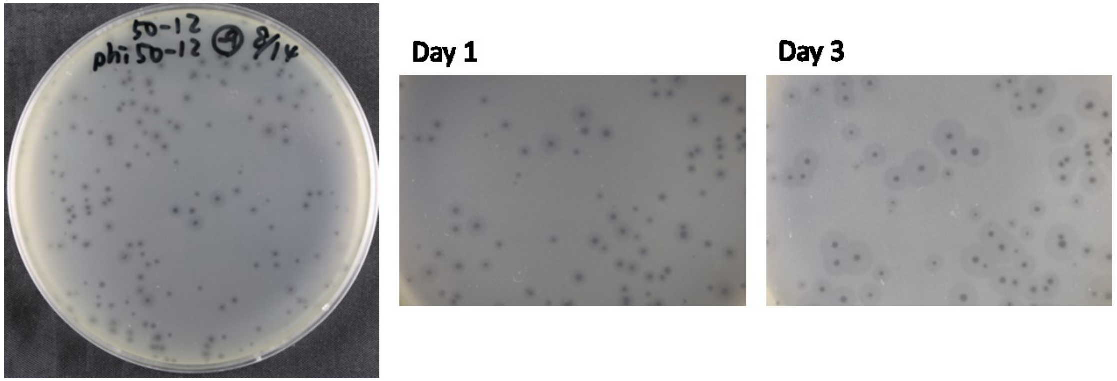

Supplementary Figure S2. Plaque morphology of phi50-12.

(A) Plaques of phi50-12 were formed on bacterial lawns of *V. owensii* GRA50-12; halo zones are observed surrounding the clear center of plaques. The halo zone area increased over days. (B) Plaques on the lawns of host cells on Day 1 and Day 3 after plating

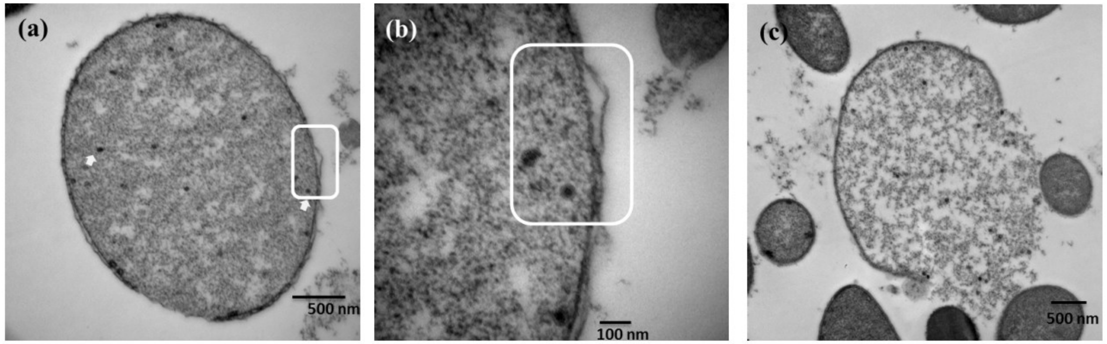

Supplementary Figure S3. Ultrathin section of transmission electron micrographs of phi50-12 infecting *V. owensii* GRA50-12.

V. owensii GRA50-12 was infected by phi50-12 with MOI=10 for 50 minutes. (A) The locations of virion release on infected cells were observable as a bulge and an irregular surface. (B) The magnification of the white box region of (A). (C) An infected cell blasting out and

releasing mature virions. Scale bars, 100 nm.

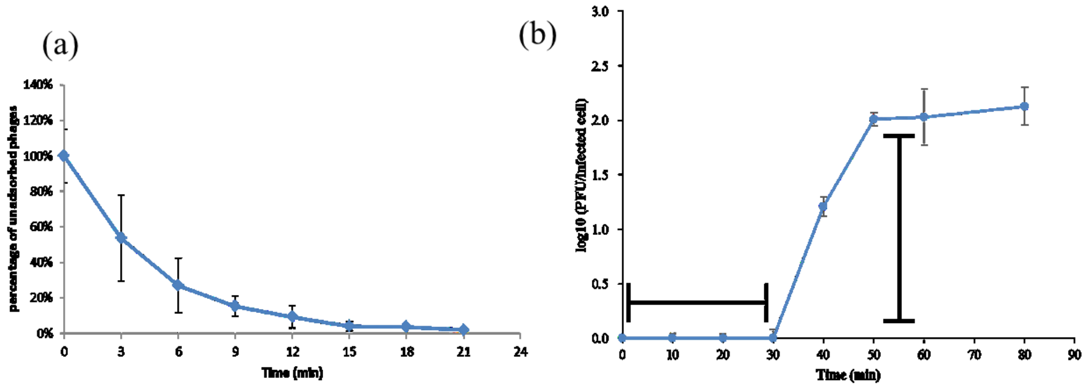

Supplementary Figure S4. Biological properties of phi50-12 (a) Phage adsorption curve for phi50-12. **(b)** One-step growth curve with host *V. owensii* GRA50-12 infected at 30 °C. Each point represents the mean of three independent experiments.

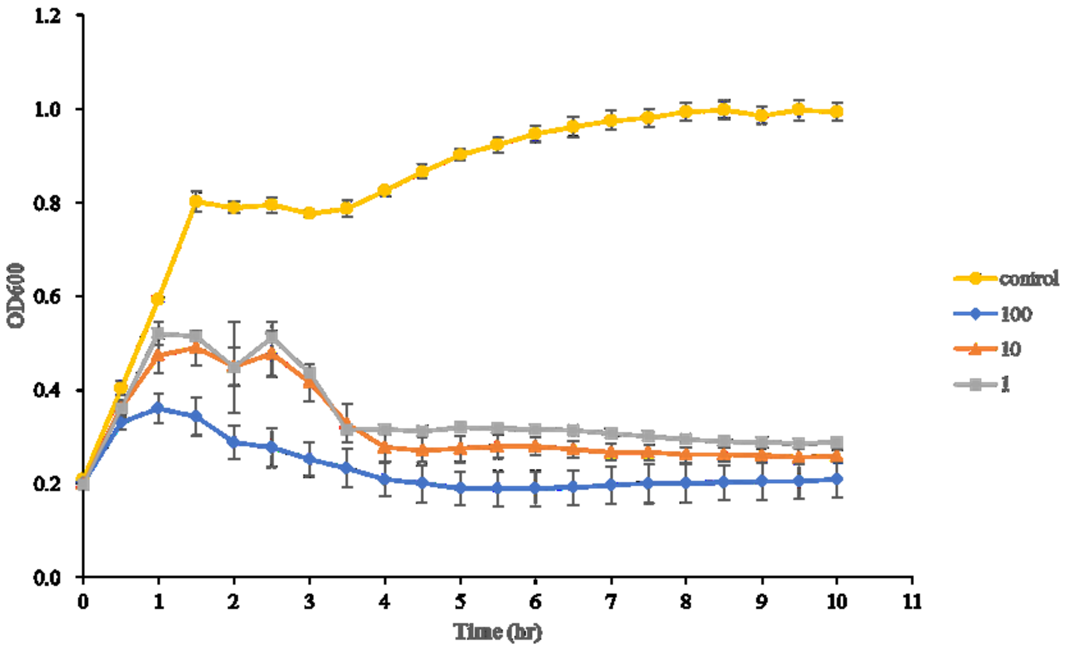

Supplementary Figure S5. In vitro lysis analysis of phi50-12 against *V. owensii* GRA50-12 at different MOIs. All values are the mean of triplicate measurements with standard deviation, and two independent experiments were performed.

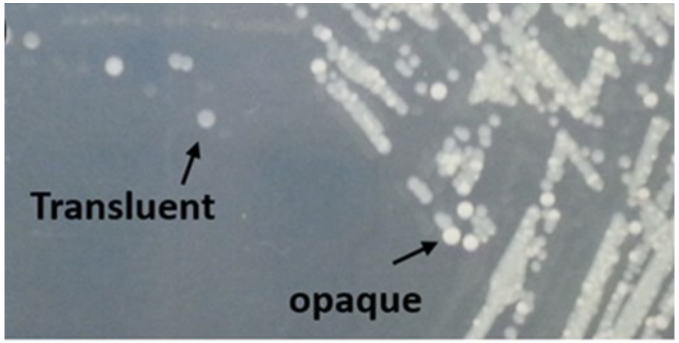

Opaque type → Translucent type

Supplementary Figure S6. Colony morphology changes when *V. owensii* GRA50-12 was challenged with phi50-12.

V. owensii GRA50-12 was growing into mid-log phase and infected with MOI=0.1 MOI of phi50-12, then cultured for 24 hours at 30℃. The overnight cultures were streaked on a TSB3S plate and incubated for 24 h at 30℃. The colony morphology changed from opaque to translucent.

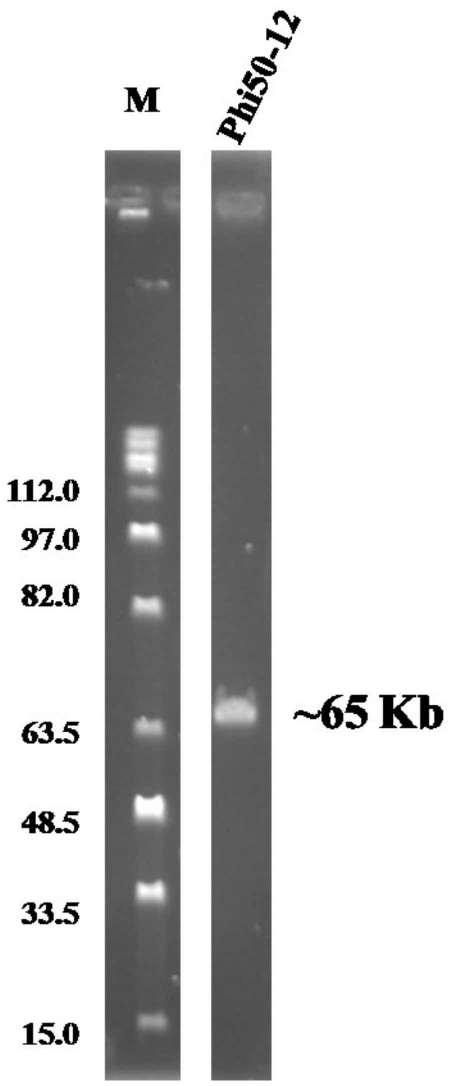

M
Phi50-12
112.0
97.0
82.0
63.5
48.5
33.5
15.0
~65 Kb

Supplementary Figure S7. Estimation of the genome size of phi50-12 using pulsed-field gel electrophoresis (PFGE). Genomic DNA of phi50-12 was isolated and the CHEF-DR III System (Bio-Rad Laboratories, Hercules, CA, USA) was used to estimate the molecular size under the following conditions: 9 °C in 0.5 × Tris-borate-EDTA buffer (pH 8.0) at 6 V/cm with pulse ramps from 3.5 to 4 s for 19.5 h. A Midrange IPFG Marker (New England Biolabs, Ipswich, MA, USA) was used as the molecular size standard. Original gel are presented in Supplementary Figure S10.

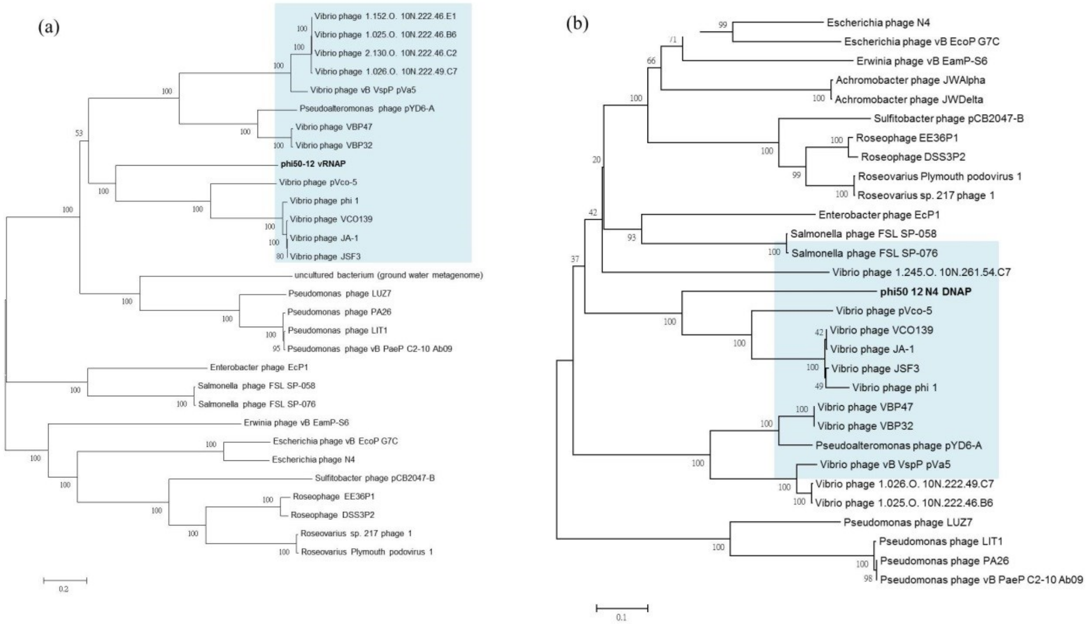

Supplementary Figure S8. Phylogenetic analysis of virion-associated RNA polymerase (vRNAP)(5A) and DNA polymerase (DNAP) (5B). The tree was generated using MEGA11 based on the Neighbor-joining method with 1000 bootstraps. This analysis involved all Vibriophages currently available in the reference database.

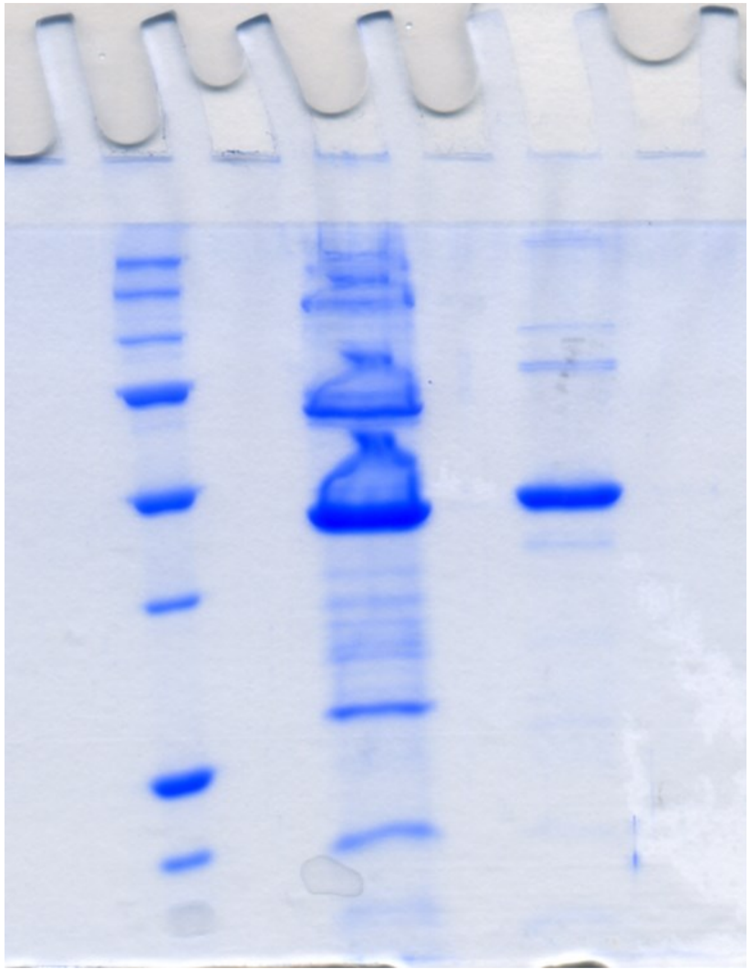

Supplementary Figure S9. The original gel of Figure 5.

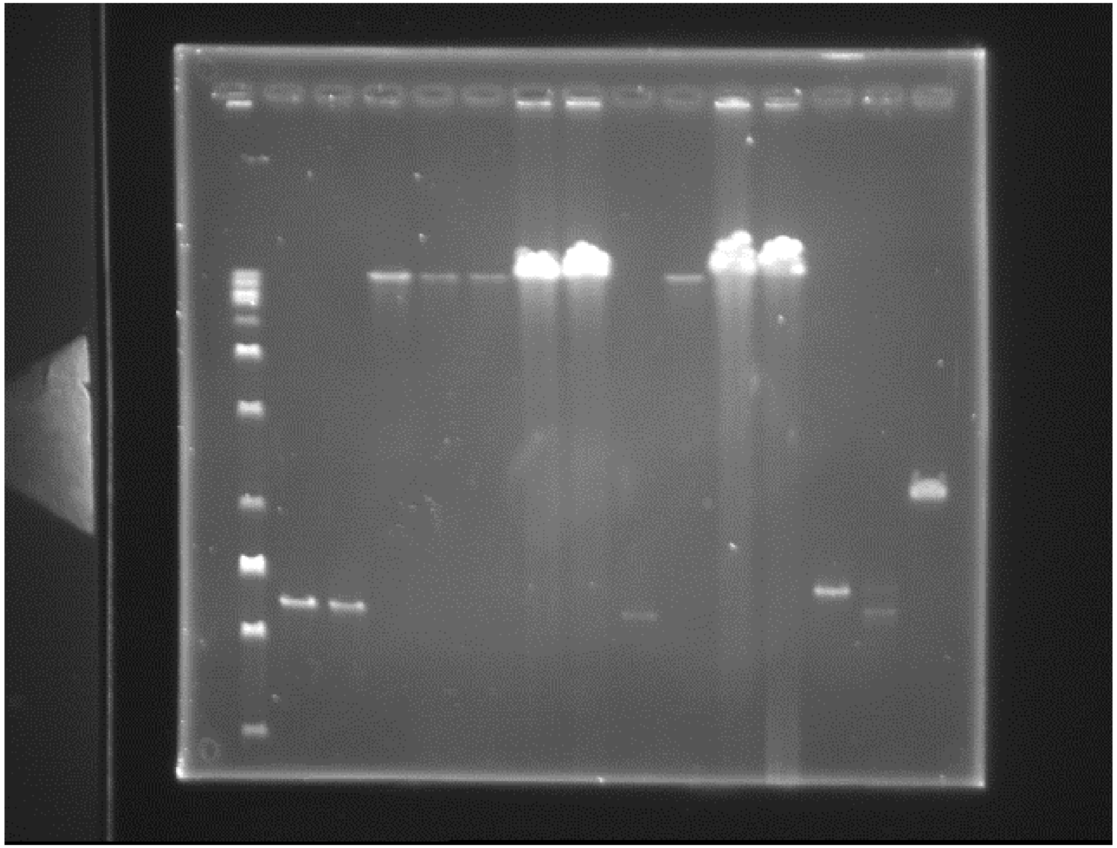

Supplementary Figure S10. The original gel of Supplementary Figure S7
